# Genetic variant of MIR4300HG is associated with progression of adolescent idiopathic scoliosis in a Chinese population

**DOI:** 10.1186/s13018-021-02455-w

**Published:** 2021-05-13

**Authors:** Yuwen Wang, Zhicheng Dai, Zhichong Wu, Zhenhua Feng, Zhen Liu, Xu Sun, Leilei Xu, Yong Qiu, Zezhang Zhu

**Affiliations:** 1 Department of Spine Surgery, Drum Tower Hospital Clinical College of Nanjing Medical University, Zhongshan Road 321, Nanjing, 210008 China; 2 Department of Spine Surgery, Drum Tower Hospital of Nanjing University Medical School, Nanjing, China

**Keywords:** Adolescent idiopathic scoliosis, MIR4300HG, Curve progression, Genetic variant

## Abstract

**Background:**

A recent genome-wide association study identified a susceptible locus in MIR4300HG gene that was associated with curve progression of adolescent idiopathic scoliosis (AIS) in the Japanese population. However, the association between the gene and curve progression in other populations remains unclear.

**Methods:**

A cohort of 1952 AIS patients and 2495 healthy controls were included in the case-control analysis. In the case-only analysis, 747 patients were assigned to the progression group and 520 patients were assigned to the non-progression group, respectively. Rs35333564 was genotyped for all the subjects. Paraspinal muscles of 76 patients were collected for the analysis of gene expression. Chi-square test and ANOVA test were used for the intergroup comparison. Pearson correlation analysis was performed to investigate the relationship between the gene expression and curve magnitude.

**Results:**

Variant rs35333564 was significantly associated with the curve severity of AIS (*p* = 0.025), but not the development of AIS (*p* = 0.418). Genotype GG was indicated by remarkably lower expression of MIR4300 (*p* = 0.020) which was significantly correlated with curve magnitude (*p* = 0.010). As a predicted target gene of MIR4300, the expression of CRTC1 was negatively correlated with MIR4300 expression (*p* = 0.012, *r* = −0.287) and positively correlated with curve severity (*p* = 0.025, *r* = 0.257).

**Conclusions:**

The association between rs35333564 and curve progression was successfully replicated in a Chinese AIS population. CRTC1 may be the target gene of MIR4300 that plays a role in the curve progression of AIS.

**Supplementary Information:**

The online version contains supplementary material available at 10.1186/s13018-021-02455-w.

## Introduction

Adolescent idiopathic scoliosis (AIS) is a complex spinal deformity that affects 2–3% of children aged 10–16 years worldwide [1]. The curves in some AIS patients with an expected residual growth of the spine may progress to larger magnitude which commonly requires spinal fusion surgery when exceeding 45° [1]. Considering the significant psychological trauma and financial burden caused by surgery, accurate prediction of curve progression can undoubtedly facilitate early intervention with individualized strategy, ultimately leading to better prognosis and reduced negative effects from excessive treatment [2]. However, conventional predictive models using clinical predictors such as skeletal immaturity, initial Cobb angle, and vertebral rotation were not so effective to be used as diagnostic criteria [3]. More reliable prognostic factors need to be identified to increase the accuracy of the predictive model.

Genetic components have been shown to play an important role in AIS curve progression, as evidenced by several zygotic twins and family studies [4–7]. It has been well documented that the combined effect of genetic variants may have better prediction for the prognosis of the complex disease [8, 9]. Ward et al. [10] developed the first DNA-based prognostic test “ScoliScore” to predict AIS curve progression in the Caucasians. However, the component SNPs of ScoliScore were not successfully replicated in the Chinese, the Canadian-French, or the Japanese population, thus implying the importance of validation study when embedding new genetic markers in the predictive model of curve progression [11–14]. Recently, Ogura et al. [15] conducted a GWAS study and identified that a functional variant rs35333564, located in the host gene of microRNA MIR4300 (MIR4300HG), was associated with progression of AIS. However, there is a lack of replication study concerning the association of MIR4300HG with curve progression in other populations. The purpose of the present study was to validate the association of MIR4300HG with the progression of AIS and to further determine the target gene of MIR4300 that may be involved in the curve progression.

## Methods

### Subject

This retrospective study was approved by the institutional review board of Drum Tower Hospital of Nanjing University Medical School (Institutional Review Board no. 2019-066-01). Informed consent was obtained from all participants and guardians. We reviewed female AIS patients who visited our scoliosis center between July 2008 and September 2019 to determine whether they were eligible to be included in this study. Healthy participants were recruited during the physical examination before university admission. The following inclusion criteria were used: (1) AIS was diagnosed by clinical and radiological examination, (2) no history of braces or other conservative treatment, and (3) with Cobb angle more than 20°. Through the Adam’s Forward Bend Test by a senior orthopedic surgeon (Y.Q), scoliosis in healthy individuals was excluded [16]. Overall, there were 1952 patients and 2495 controls included in the case-control analysis.

For the case-only analysis, we divided AIS patients into the progression and non-progression groups. Patients with Cobb angle more than 50° and Risser grade less than 3 were assigned to the progression group, and patients with Cobb angle less than 30° and Risser grade more than 3 at the final follow-up were assigned to the non-progression group. Ultimately, 747 patients were assigned to the progression group and 520 patients were assigned to the non-progression group, respectively. At each visit, standing posteroanterior radiographs of the spine were obtained and demographic characteristics were recorded, including Cobb angle of the main curve, Risser sign, BMI, and menarche age.

### Genotyping of the target SNP

Blood samples from each patient were collected for the extraction of genomic DNA following standard methods (Qiagen K.K., Tokyo, Japan). We used the TaqMan SNP genotyping method to genotype rs35333564 of MIR4300HG on the ABI PRISM 7900HT sequence detection system (Applied Biosystems, Foster City, CA). To verify the reliability of genotyping results, 20% samples were randomly selected. The reproducible rate was 100%.

### Expressions of the genes in the paraspinal muscles

The paraspinal muscles of the proximal vertebral segments of 76 patients were collected during the operation. Total RNA was isolated using Trizol reagent (Invitrogen, Carlsbad, CA) in accordance with the protocol of the manufacturer. To explore the expression level of MIR4300, complementary DNA was synthesized using a specific primer: 5′-CCTGTTGTCTCCAGCCACAAAAGAGCACAATATTTCAGGAGACAACAGGGAAGTAG-3′. Quantitative polymerase chain reaction (PCR) analysis used the following primers: forward 5′-CGGGCTGGGAGCTGGA-3′, reverse 5′-CAGCCACAAAAGAGCACAAT-3′ for MIR4300; forward 5′-CTCGCTTCGGCAGCACA-3′, reverse 5′-AACGCTTCACGAATTTGCGT-3′ for the endogenous control gene U6. Moreover, to further analyze the target gene of MIR4300 that exerts effect on the curve progression of AIS, a list of 55 genes was predicted using an on-line tool (TargetScan Human7.0 and miRWalk2.0 database) and the primers were designed for each gene as shown in Additional file 1. The gene expression was detected using a SYBR green-based real-time quantitative PCR assay (Toyobo Co., LDT, Osaka, Japan) as formerly described [17]. Melting point curve analysis was performed after cycling to ensure the quality of qPCR products. The 2^−ΔΔCt^ method was used to determine the quantitative measurements.

### Statistical analysis

Statistical analysis was performed by using SPSS software (version 20.0; SPSS Inc., Chicago, IL). The Hardy-Weinberg equilibrium (HWE) was tested in the AIS patients and healthy controls, using a *χ*^2^ goodness-of-fit test. Intergroup comparisons of the genotype and allele frequency were analyzed using the chi-square test. Minor allele G was used as a reference to calculate the odds ratio (OR). The ANOVA test was used to compare with expression of MIR4300 among different genotypes of rs35333564. The Pearson correlation analysis was performed to investigate the relationship between the expression level of MIR4300 and the predicted target genes, as well as the relationship between the gene expression level and the curve severity. *p* < 0.05 was considered to be statistically significant.

## Results

### Clinical characteristics of the participants

In the case-control analysis, the mean age was 13.6 ± 2.8 years for the AIS patients (*n*= 1952) and 19.2 ± 3.6 years for healthy controls (*n*= 2495). For AIS patients, the mean curve magnitude was 36.8 ± 3.2° (range 22–66°), the mean BMI was 17.5 ± 3.4 kg/m^2^ (range, 16.3–24.6 kg/m^2^), and the mean menarche age was 12.3 ± 1.8 years (range, 10.2–15.3 years), respectively. There were 1218 (62.4%) cases with main thoracic curve, 476 (24.3%) cases with double major curve, and 258 (13.3%) cases with major lumbar curves, respectively.

As shown in Table 1, for the case-only analysis, the two groups were matched in terms of initial age, menarche age, and BMI (13.2 ± 2.4 years vs. 13.0 ± 2.3 years, *p* = 0.129 for initial age; 13.3 ± 1.3 years vs. 13.1 ± 1.2 years, *p* = 0.128 for menarche age; 18.2 ± 2.3 kg/m^2^ vs. 17.9 ± 2.4 kg/m^2^ for BMI, *p* = 0.152). There were remarkably higher Cobb angle and less initial Risser sign in the progression group than in the non-progression group (65.8° ± 9.7° vs. 25.9° ± 3.5°, *p* <0.001 for Cobb angle; 0.6 ± 0.9 vs. 4.3 ± 0.5, *p* < 0.001 for Risser sign).

**Table 1:** Baseline characteristics of the subjects enrolled in the case-only analysis

	Progression group	Non-progression group	*p*-value
Initial age (years)	13.2 ± 2.4	13.0 ± 2.3	0.129
Menarche age (years)	13.3 ± 1.3	13.1 ± 1.2	0.128
BMI (kg/m^2^)	18.2 ± 2.3	17.9 ± 2.4	0.152
Cobb angle (°)	65.8 ± 9.7	25.9 ± 3.5	<0.001
Initial Risser sign	0.6 ± 0.9	4.3 ± 0.5	<0.001

For the 76 patients included in the gene expression analysis, the average curve magnitude was 64.1° ± 6.7° (range, 53 to 78°). The mean age and Risser grade were 13.2 ± 1.6 years and 4.3 ± 0.7, respectively.

### Association of the rs35333564 with the onset of AIS

Genotype frequency in the patients and the healthy controls revealed no significant departure from the Hardy-Weinberg equilibrium (*p* > 0.05). The distribution of genotype and allele frequency of the subjects is summarized in Table 2. There was no significant difference regarding genotype frequency (3.2% vs. 2.8%, *p* = 0.418) or the minor allele frequency (16.6% vs. 15.6%, *p* = 0.181) between the two groups.

**Table 2:** Distribution of the genotype and allele frequency of rs35333564 in AIS patients and healthy controls

	Genotype			*p*	Allele		*p*	Odds ratio (95% CI)	*p*^a^
	GG	AG	AA		G	A			
				0.418			0.181	1.08 (0.96–1.21)	
Patients (*n*=1952)	62 (3.20%)	524 (26.8%)	1366 (70.0%)		648 (16.6%)	3256 (83.4%)			0.179
Controls (*n*=2495)	69 (2.80%)	638 (25.6%)	1788 (71.6%)		776 (15.6%)	4214 (84.4%)			0.187

*CI* confidential interval

*p*^a^ indicates the *p* value of the HWE test

### Association of rs35333564 with the curve progression

**Fig. 1:**
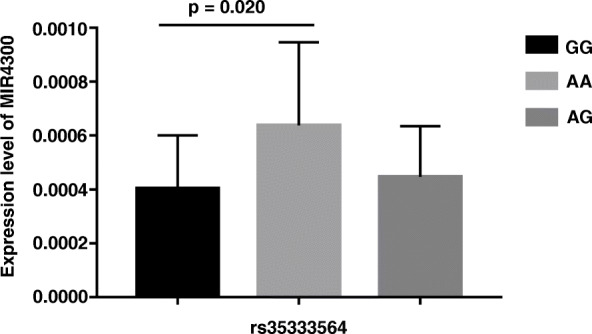
Relationship between the genotype of rs35333564 and MIR4300 expression. Patients with genotype GG had significantly lower MIR4300 expression than those with genotype AA (0.000403 ± 0.000188 vs. 0.000637 ± 0.000306, *p* = 0.020)

As shown in Table 3, there was significant difference between curve progression group and non-progression group regarding both genotype frequency (3.1% vs. 1.3%, *p* = 0.025) and minor allele frequency (17.5% vs. 13.7%, *p* = 0.011) of rs35333564 in MIR4300 gene. Furthermore, the curve in adolescent idiopathic patients with an allele G will be prone to progression, with an OR value of 1.339 (95% CI = 1.072–1.671). For tissue expression analysis, there were 11 cases with genotype GG, 15 cases with genotype GC, and 50 cases with genotype AA. Patients with genotype GG were found to have significantly lower MIR4300 expression than those with genotype AA (0.000403 ± 0.000188 vs. 0.000637 ± 0.000306, *p* = 0.020) (Fig. 1).

**Table 3:** Distribution of the genotype and allele frequency of rs35333564 in the progression group and non-progression group

	Genotype			*p*	Allele		*p*	Odds ratio (95% CI)
	GG	AG	AA		G	A		
				0.025			0.011	1.34 (1.07–1.67)
Progression group (*n*=747)	23 (3.1%)	215 (28.8%)	509 (68.1%)		261 (17.5%)	1233 (82.5%)		
Non-progression group (*n*=520)	7 (1.3%)	128 (24.6%)	385 (74.1%)		142 (13.7%)	898 (86.3%)		

*CI* confidential interval

### Relationship between gene expression level and clinical features of the patients

**Fig. 2:**
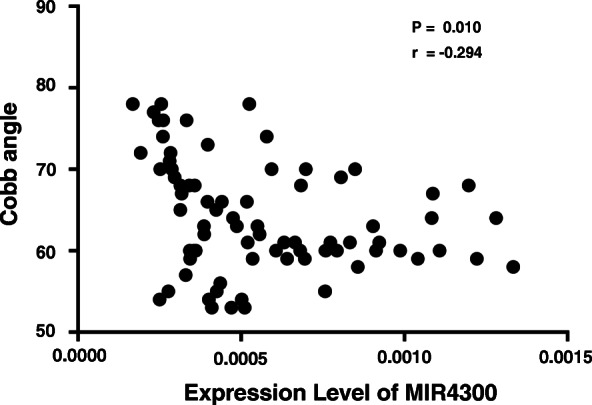
The correlation between MIR4300 expression and Cobb angle in AIS patients. The expression of MIR4300 was significantly correlated with Cobb angle in AIS patients

**Fig. 3:**
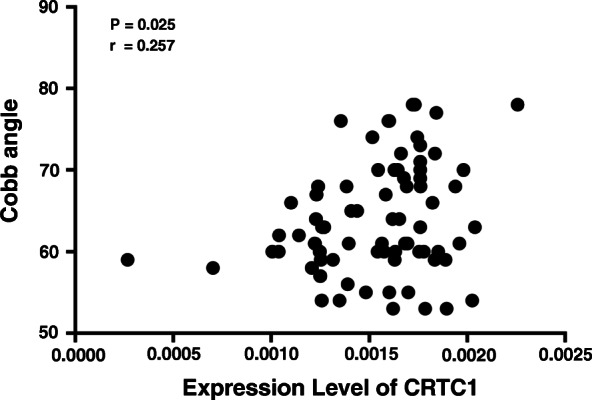
The correlation between CRTC1 expression and Cobb angle in AIS patients. The expression of CRTC1 was significantly correlated with Cobb angle in AIS patients

The expression of MIR4300 was significantly correlated with curve magnitude (*r* = −0.294, *p* = 0.01) (Fig. 2). Among the 55 predicted target genes, the expression level of CRTC1 was found inversely correlated with that of MIR4300 with statistical significance (*r* = −0.287, *p* = 0.012) (Table 4). Moreover, there was a positive correlation between the expression of CRTC1 and curve magnitude (*r* = 0.257, *p* = 0.025) (Fig. 3). No significant correlation was found between the expression of CRTC1 and other clinical parameters including BMI (*r* = 0.100, *p* = 0.390) or menarche age (*r* = 0.076, *p* = 0.516).

**Table 4:** The correlation between MIR4300 expression and CRTC1 expression in AIS patients. The expression level of CRTC1 was found inversely correlated with that of MIR4300

Gene	CRTC1	MIR4300	*p*-value	*r*
Mean expression	0.00155 ± 0.00032	0.00057 ± 0.00029	0.012	−0.287

## Discussion

Most of previously reported genetic markers predicting curve progression in AIS patients, including SNPs in IGF1 and ESR2, were not successfully replicated by other studies [18, 19]. Therefore, it is necessary to verify the association of reported locus with AIS progression in different ethnic populations. Ogura et al. [15] reported a functional variant rs35333564 located in MIR4300HG was associated with the progression of AIS in the Japanese population. Based on a large cohort of subjects, our study successfully validated that rs35333564 of MIR4300HG was associated with AIS progression in a Chinese population. Moreover, allele G in rs35333564 could significantly add to the risk of curve progression with an OR of 1.339, which is slightly lower than that reported by Ogura et al. [15]. In addition, we also explored the relationship of the locus with the onset of AIS by genotyping in both AIS patients and normal controls, while no significant association was observed. To summarize, rs35333564 of MIR4300HG may not be the trigger for the development of AIS but more likely the fuel driving curve progression.

To explore the potential functional role of rs35333564, for the first time, we evaluated the relative expression level of MIR4300 in the paraspinal muscles among patients with different genotypes of rs35333564. Patients with genotype GG were found to have remarkably lower tissue expression of MIR4300 than those with genotype AA. This finding was in line with the luciferase assay performed by Ogura et al. [15], which showed that allele G can remarkably lower the transcriptional activity of the pGL3 promoter vector. Interestingly, the tissue expression level of MIR4300 was found significantly correlated with curve severity in the current study. Taken together, it was plausible that rs35333564 may play a functional role in the curve progression by down-regulating the tissue expression of MIR4300.

To date, the function of MIR4300 remains unknown. As reported in the study of Ogura et al. [15], 55 genes were predicted to be the potential target of MIR4300. Through the expression analysis of these 55 genes, we further identified that CRTC1 was remarkably correlated with MIR4300 expression as well as the curve severity. Berdeaux et al. [20] reported that CREB-CRTCs are key cAMP effectors, which promote muscle adaptation by activating transcription programs, thereby increasing skeletal muscle performance. Considering histochemical and physiological abnormalities in muscles of patients with idiopathic scoliosis [21], we suggested that CRTC1 may be the target gene of MIR4300 that may function in the curve progression. Further studies are warranted to investigate the underlying mechanism by in vivo and intro experiments.

In the present study, we validated the association between genetic variants of MIR4300 with curve progression by genotyping in a relatively large population. Moreover, we identified CRTC1 as the potential target gene of MIR4300 and found that the expression level of MIR4300 and CRTC1 in paraspinal muscles was significantly correlated with curve magnitude. Thus, we speculate that the miRNA encoded by MIR4300 were involved in the progression via regulating the expression of CRTC1. There are two limitations in the current study that need to be mentioned. Firstly, we only evaluated MIR4300 expression in muscle samples as few patients agreed to donate other tissues; further studies need to include more AIS-related tissues for the evaluation of MIR4300 expression. Secondly, only AIS patients who underwent surgical intervention (Cobb angle exceeds 50°) were enrolled in the expression analysis which could bias the results. Therefore, the association of MIR4300 expression with curve severity should be interpreted cautiously.

## Conclusions

The association between rs35333564 and curve progression was successfully replicated in a Chinese AIS population. Allele G can add to the risk of curve progression possibly through the down-regulation of MIR4300. CRTC1 may be the target gene of MIR4300 that plays a role in the curve progression of AIS. Further studies are warranted to explore the molecular mechanism underlying the effect of MIR4300 on curve progression in AIS patients.

## Supplementary Information

**Additional file 1: Table S1.** Primers of the 55 predicted target genes of MIR4300.

## Data Availability

The data generated or analyzed during this study are included in this published article [and its supplementary information files].

## Abbreviation

AIS: Adolescent idiopathic scoliosis
