# Supplementary material for: Genetic variant of MIR4300HG is associated with progression of adolescent idiopathic scoliosis in a Chinese population
Source: J Orthop Surg Res. 2021 May 13;16:311. doi: 10.1186/s13018-021-02455-w (PMC8117547; doi:10.1186/s13018-021-02455-w)
Supplement: Supplementary file 1 — Additional file 1: Table S1. Primers of the 55 predicted target genes of MIR4300. [file 13018_2021_2455_MOESM1_ESM.docx]

**Additional Table 1 Primers of the 55 predicted target genes of MIR4300**

| **Gene** | **Primer name** | **Primer sequence (5′-3′)** | **Tm (°C)** |
| --- | --- | --- | --- |
| AARS | AARS-F | ATTGATGAGCCCCGACGAAG | 60.2 |
|  | AARS-R | AGTTCCGCAGGTGCCTTG | 60.3 |
| ATAD3A | ATAD3A-F | GCGAGCCACCGAGAAGATAAG | 62.6 |
|  | ATAD3A-R | TGGACCATCTCATTGATGCGG | 62.2 |
| ATAD3B | ATAD3B-F | CCTGAGCAGTTCGACTGTG | 60.1 |
|  | ATAD3B-R | GCGCCGTTTTCCTTCTGTG | 62.0 |
| BTN2A2 | BTN2A2-F | GGGCCAGCTAATCCCATCC | 61.9 |
|  | BTN2A2-R | GGTGATTCTTCCCCGGTACTC | 61.6 |
| C11orf49 | C11orf49-F | CTACCGGACTACGAGTATCTGG | 60.6 |
|  | C11orf49-R | TGTGTTCCCTGGCATACACTG | 62.0 |
| C18orf25 | C18orf25-F | AGAGAGTGACAAGCCTACAACT | 60.4 |
|  | C18orf25-R | AGGTGACCACTAGAGGAAGGG | 62.1 |
| C9orf114 | C9orf114-F | GCCGCCTATTTGGACACCTA | 59.8 |
|  | C9orf114-R | ACTCAATCCTTTGGCCGTGT | 59.9 |
| C9orf3 | C9orf3-F | CACATGGCTGGATGCGGATA | 60.3 |
|  | C9orf3-R | ACCGCACCTGGCCTTTATTA | 59.4 |
| CAV2 | CAV2-F | AAGACCTGCCTAATGGTTCTGC | 62.3 |
|  | CAV2-R | CTCGTACACAATGGAGCAATGAT | 60.7 |
| CDH6 | CDH6-F | CTCGGGAAGGCAAGTAAAGC | 60.7 |
|  | CDH6-R | TGAGAGTGCAGATGTTCGACC | 61.8 |
| CHD8 | CHD8-F | AAGCAAATCGGATTGTAGCAGA | 58.4 |
|  | CHD8-R | GGCAACTCGTCCTCATTTAAGA | 58.4 |
| CHRNA4 | CHRNA4-F | GGAGGGCGTCCAGTACATTG | 62.3 |
|  | CHRNA4-R | GAAGATGCGGTCGATGACCA | 62.0 |
| CLMN | CLMN-F | CTCATCGGGCAGATTAGCGAC | 62.6 |
|  | CLMN-R | TCCATCGTGTAAAGGTCCTCT | 62.0 |
| COPG1 | COPG1-F | TCTTTGCCATGACCAAGCTCT | 61.7 |
|  | COPG1-R | GGCCCCGGTAGTTGTCTTC | 62.0 |
| CRTC1 | CRTC1-F | TCAGTGGACAAACACGGACG | 62.3 |
|  | CRTC1-R | CAGGGCGGAGTCAGAATTG | 60.2 |
| CSF1 | CSF1-F | AGACCTCGTGCCAAATTACATT | 60.0 |
|  | CSF1-R | AGGTGTCTCATAGAAAGTTCGGA | 60.5 |
| CSNK1D | CSNK1D-F | GTGTAAAGGCTACCCTTCCGA | 61.2 |
|  | CSNK1D-R | TACGAGTAGTCAGGCTTGTCG | 61.0 |
| CST1 | CST1-F | CCTGTGCCTTCCATGAACAGCC | 59.1 |
|  | CST1-R | GGGTGGTGGCTGGTGCCAATG | 66.7 |
| CST2 | CST2-F | AGGAGGACAGGATAATCGAGGG | 62.2 |
|  | CST2-R | CGCTGTACCCGCTCATCATT | 62.4 |
| CST4 | CST4-F | CCTCTGTGTACCCTGCTACTC | 61.0 |
|  | CST4-R | CTTCGGTGGCCTTGTTGTACT | 62.3 |
| CST5 | CST5-F | TGCTGCTGACTGCCTTGATG | 61.0 |
|  | CST5-R | CACACTGCACACTCTTGTCAT | 58.8 |
| CST9L | CST9L-F | CAATGTCATGGCTCGTTACCT | 60.1 |
|  | CST9L-R | GGCATAGTAGTCCTTGCTCTGT | 61.2 |
| CXorf38 | CXorf38-F | CAGGTGTGCGCTGAATGGA | 62.6 |
|  | CXorf38-R | GGTCCTTGTTTGTCTGCTAGTC | 60.6 |
| DDX39A | DDX39A-F | GCAGATTGAGCCTGTCAACG | 61.3 |
|  | DDX39A-R | AGACCACCGAAGAACACAGAC | 61.7 |
| DOK1 | DOK1-F | TCCCAGGACTCCCTATACTCA | 58.5 |
|  | DOK1-R | GCTCCCGAGGCAGATCATAAA | 59.9 |
| EBP | EBP-F | CACAGGGGTCTTAGTCGTGAC | 60.0 |
|  | EBP-R | CCAGGTGAATGAACCCACACA | 60.5 |
| EFS | EFS-F | TGCTCCCTATGATGTGCCTCT | 60.7 |
|  | EFS-R | TGACGCTCGTTTCAGGTTGG | 60.9 |
| EPHB1 | EPHB1-F | CGGAATGGTTTTTACCTCGCT | 58.9 |
|  | EPHB1-R | ATGTGCCCCGAGCAATCAC | 60.8 |
| ESR1 | ESR1-F | CCCACTCAACAGCGTGTCTC | 61.0 |
|  | ESR1-R | CGTCGATTATCTGAATTTGGCCT | 59.2 |
| FAM89B | FAM89B-F | ACGAGTCAATCCAGGACTACA | 58.2 |
|  | FAM89B-R | AATGGAGGGAGGATGACAGGT | 60.3 |
| FBRSL1 | FBRSL1-F | GGGGAGAGGCTACTCTTGTGA | 60.6 |
|  | FBRSL1-R | GGGCCTTTATCGGTTCCCG | 60.5 |
| FCRL6 | FCRL6-F | CTGTCTGGCTGTACCTCCAAG | 60.1 |
|  | FCRL6-R | CATCCCTGACATCGCAGAGTC | 60.5 |
| FILIP1 | FILIP1-F | CCCTGGAGTTATCCAAAGAAGAC | 58.5 |
|  | FILIP1-R | CGCAGACCCGTAATGAGCC | 60.9 |
| FZR1 | FZR1-F | CTGGAAGTACCCTCCCTGA | 59.9 |
|  | FZR1-R | GAGCACAGACACAGACTCCC | 60.0 |
| GPC5 | GPC5-F | TCTAATATCTCGAAATGCGGCTG | 58.8 |
|  | GPC5-R | GGCCATGTTCCTGTAGGTACTG | 60.4 |
| GRIPAP1 | GRIPAP1-F | GGACAAACAACTACCAGCTTTCA | 59.4 |
|  | GRIPAP1-R | GCGACCTTCTGTCGAAGACT | 59.8 |
| KDM5C | KDM5C-F | GGGTCCGACGATTTCCTACC | 59.9 |
|  | KDM5C-R | ATGCCCGATTTCTCTGCGATG | 61.1 |
| KIF6 | KIF6-F | ATGGTGAAGCAGACTATCCAGA | 58.6 |
|  | KIF6-R | ACGGTCTCTTGGTTTGCATCC | 60.9 |
| KLHL33 | KLHL33-F | GCGTGGGACTGGTACGAAC | 60.7 |
|  | KLHL33-R | GGGTGTTGGAGTGACTGTAGA | 59.0 |
| MAPK8IP3 | MAPK8IP3-F | GTGTACCAGGACGACTACTGC | 60.1 |
|  | MAPK8IP3-R | GCACCGAGTCTAGGTTCTCCA | 61.2 |
| MED28 | MED28-F | GGCGCTCCTAGACCTTCCA | 63.0 |
|  | MED28-R | GTGCCATTGACATAGTCCTGAC | 60.7 |
| MPHOSPH6 | MPHOSPH6-F | GCACTGGTACTTGGATTTGCC | 59.8 |
|  | MPHOSPH6-R | TGCTTTGTGCTTAGCATTCATCT | 59.2 |
| MRPL15 | MRPL15-F | GTTTCAGACGCCAGTATAAGCC | 59.4 |
|  | MRPL15-R | ACTAGGATCAACACGACCCAAAT | 59.7 |
| MSMB | MSMB-F | CTGGGCAGCGTTGTGATCTT | 61.0 |
|  | MSMB-R | CCTGGAACTCCCTCATTAGGTAT | 58.8 |
| MUC1 | MUC1-F | TGCCGCCGAAAGAACTACG | 60.7 |
|  | MUC1-R | TGGGGTACTCGCTCATAGGAT | 59.9 |
| NCOA5 | NCOA5-F | ATGAATACGGCTCCATCAAGACC | 60.5 |
|  | NCOA5-R | ACTTCCTCGAATTGGGGATCG | 59.9 |
| NFYB | NFYB-F | ATGACAATGGATGGTGACAGTTC | 59.0 |
|  | NFYB-R | CTAGCCACGTTTGCTATTGGA | 58.4 |
| NGRN | NGRN-F | ATGGCGGTTACCCTGAGTCT | 60.6 |
|  | NGRN-R | GGAATCGGATTGCTTGTTTCTGT | 59.8 |
| NUCKS1 | NUCKS1-F | GGCCTGTCAGAAATAGGAAGGT | 59.8 |
|  | NUCKS1-R | TTTAGCTTCTCGGGGAGATGAT | 58.7 |
| PDXK | PDXK-F | GATTTGAGATTGACGCGGTGA | 59.0 |
|  | PDXK-R | CCCTCGTATAACCTGTGAGCAC | 60.5 |
| PLAUR | PLAUR-F | TGTAAGACCAACGGGGATTGC | 60.6 |
|  | PLAUR-R | AGCCAGTCCGATAGCTCAGG | 61.1 |
| RASA4 | RASA4-F | CAGCCGGGACGACGTTATC | 60.6 |
|  | RASA4-R | CCACCCGCTGAAACCCTTAG | 60.7 |
| RASGRP3 | RASGRP3-F | CTCTGCATGTATCGAAATGCCA | 59.1 |
|  | RASGRP3-R | CTACTTCCCGAAATTCCTCAGTC | 59.6 |
| RHBG | RHBG-F | CTCCATGACTATCCACACCTTTG | 58.8 |
|  | RHBG-R | GAGGTCTGAATGGTAGACGGA | 58.6 |
| RHOD | RHOD-F | TTTGAGCGGTACATGGTCAAC | 58.9 |
|  | RHOD-R | AGGCGGTCATAGTCATCTTGC | 60.2 |
| GAPDH  (Endogenous control) | GAPDH-F | GAGTCAACGGATTTGGTCGT | 58.2 |
|  | GAPDH-R | TTGATTTTGGAGGGATCTCG | 55.1 |
